# SIMIFF study: Italian fungal registry of mold infections in hematological and non-hematological patients

**DOI:** 10.1007/s15010-013-0539-3

**Published:** 2013-10-23

**Authors:** M. T. Montagna, G. Lovero, C. Coretti, D. Martinelli, M. Delia, O. De Giglio, M. Caira, F. Puntillo, D. D’Antonio, M. Venditti, V. Sambri, F. Di Bernardo, A. Barbui, G. Lo Cascio, E. Concia, M. Mikulska, C. Viscoli, N. Maximova, A. Candoni, S. Oliveri, G. Lombardi, L. Pitzurra, M. Sanguinetti, R. Masciari, T. Santantonio, S. Andreoni, F. Barchiesi, P. Pecile, C. Farina, P. Viale, G. Specchia, G. Caggiano, L. Pagano

**Affiliations:** 1 Hygiene Section, Department of Biomedical Science and Human Oncology, University of Bari, Piazza Giulio Cesare 11, 70124 Bari, Italy; 2 Hygiene Section, Department of Medical and Surgical Sciences, University of Foggia, Via Gramsci 89-91, 71100 Foggia, Italy; 3 Hematology Section, Department of Emergency and Organ Transplantation, University of Bari, Piazza Giulio Cesare 11, 70124 Bari, Italy; 4 Hematology Institute, University Cattolica S. Cuore, Largo Francesco Vito 1, 00168 Rome, Italy; 5 Anesthesia and Intensive Care Unit, Department of Emergency and Organ Transplantation, University of Bari, Piazza Giulio Cesare 11, 70124 Bari, Italy; 6 Clinical Microbiology and Virology, Hospital Spirito Santo, Via Fonte Romana 8, 65100 Pescara, Italy; 7 Department of Public Health and Infectious Diseases, University La Sapienza, Piazzale Aldo Moro 5, 00185 Rome, Italy; 8 Clinical Microbiology Unit, University Hospital St. Orsola, Via Pietro Albertoni 15, 40138 Bologna, Italy; 9 Laboratory of Clinical Microbiology, ARNAS General Hospital Civico, Piazzale Liotti 4, 90127 Palermo, Italy; 10 SC Microbiology and Virology, Hospital Città della Salute e della Scienza, Corso Bramante 88, 10126 Turin, Italy; 11 Microbiology and Virology Unit, Department of Pathology and Diagnostic, University of Verona, Piazzale A. Scuro 10, 37134 Verona, Italy; 12 Department of Pathology, University of Verona, Piazzale A. Scuro 10, 37134 Verona, Italy; 13 Infectious Diseases Division, Department of Health Sciences, University Hospital IRCCS San Martino, Largo Rosanna Benzi 10, 16132 Genoa, Italy; 14 Oncohematology, Institute for Maternal and Child Health, IRCCS Burlo Garofolo, Via dell’Istria 65/1, 34137 Trieste, Italy; 15 Division of Hematology and Bone Marrow Transplantation, University of Udine, Via Palladio 8, 33100 Udine, Italy; 16 Department of Biomedical Science, University of Catania, Piazza dell’Università 2, 95124 Catania, Italy; 17 Microbiology and Virology Laboratory, Hospital Niguarda-Ca’ Granda, Piazza Ospedale Maggiore 3, 20162 Milan, Italy; 18 Department of Experimental Medicine, University of Perugia, Piazza Università 1, 06123 Perugia, Italy; 19 Department of Experimental Medicine, Institute of Microbiology, University Cattolica S. Cuore, Largo Francesco Vito 1, 00168 Rome, Italy; 20 Microbiology and Virology Laboratory, A.O. Pugliese Ciaccio, Via Vinicio Cortese 25, 88100 Catanzaro, Italy; 21 Clinic of Infectious Diseases, University of Foggia, Via Gramsci 89-91, 71100 Foggia, Italy; 22 Microbiology and Virology Laboratory, AOU Maggiore della Carità, Corso Mazzini 18, 28100 Novara, Italy; 23 Clinic of Infectious Diseases, Department of Biomedical Sciences and Public Health, University of Marche, Piazza Roma 22, 60121 Ancona, Italy; 24 Microbiology Laboratory, Hospital Careggi, Viale Morgagni, 50134 Florence, Italy; 25 Microbiology Institute, Hospital San Carlo Borromeo, Via Pio II 3, 20147 Milan, Italy; 26 Infectious Diseases Unit, Department of Medical and Surgical Sciences, University of Bologna, Via Zamboni 33, 40126 Bologna, Italy

**Keywords:** Filamentous fungal infections, Italian survey, Hematological patients, Non-hematological patients

## Abstract

**Purpose:**

We compared the risk factors, the diagnostic tools and the outcome of filamentous fungal infections (FFIs) in hematological patients (HAEs) and non-hematological patients (non-HAEs).

**Methods:**

Prospective surveillance (2009–2011) of *proven* and *probable* FFIs was implemented in 23 Italian hospitals.

**Results:**

Out of 232 FFIs, 113 occurred in HAEs and 119 in non-HAEs. The most frequent infection was invasive aspergillosis (76.1 % for HAEs, 56.3 % for non-HAEs), and the localization was principally pulmonary (83.2 % for HAEs, 74.8 % for non-HAEs). Neutropenia was a risk factor for 89.4 % HAEs; the main underlying condition was corticosteroid treatment (52.9 %) for non-HAEs. The distribution of *proven* and *probable* FFIs was different in the two groups: *proven* FFIs occurred more frequently in non-HAEs, whereas *probable* FFIs were correlated with the HAEs. The sensitivity of the galactomannan assay was higher for HAEs than for non-HAEs (95.3 vs. 48.1 %). The overall mortality rate was 44.2 % among the HAEs and 35.3 % among the non-HAEs. The etiology influenced the patient outcomes: mucormycosis was associated with a high mortality rate (57.1 % for HAEs, 77.8 % for non-HAEs).

**Conclusions:**

The epidemiological and clinical data for FFIs were not identical in the HAEs and non-HAEs. The differences should be considered to improve the management of FFIs according to the patients’ setting.

## Introduction

In recent years, the epidemiology of fungal infections has changed. Recent findings indicate an increasing number of filamentous fungal infections (FFIs), most likely due to the widespread usage of fluconazole prophylaxis for *Candida albicans* control [1, 2]. FFIs occur mainly among patients with hematological malignancies, especially during prolonged neutropenia, and in recipients of hematopoietic stem cell transplants (HSCTs) [3]. However, in recent years, the spectrum of high-risk patients has expanded. FFIs have also been recognized as an emerging opportunistic infection in patients with chronic obstructive pulmonary disease (COPD) [4] or connective tissue diseases requiring corticosteroid therapy, in solid cancer patients [5], HIV patients [6], ICU patients [7, 8] and patients treated with new immunosuppressive agents [9]. Recently, an increasing number of cases associated with chronic lymphoproliferative disorders has been reported [10, 11]. In these non-conventional hosts, FFIs are perceived as less of a concern; thus, the diagnosis is frequently made at a more advanced stage or at autopsy [12–14].

Regarding these epidemiological challenges, several studies report a wide distribution of fungal pathogens that reflects marked differences among the patient characteristics and prevention/treatment protocols, as well as environmental characteristics [15]. Indeed, although most FFIs are attributed to the *Aspergillus* genus, other less common molds, such as *Mucorales*, *Fusarium* and *Scedosporium* spp, are increasingly reported [16–18].

The aim of this study was to assess the epidemiology, diagnosis and outcome of FFIs in hematological patients (HAEs) and non-hematological patients (non-HAEs) and to analyze the possible differences between these two groups.

## Patients and methods

### Study design

The Italian Society of Medical Mycology (Federazione Italiana di Micopatologia Umana e Animale, FIMUA) conducted a nationwide FFI surveillance (Sorveglianza Italiana Multicentrica delle Infezioni da Funghi Filamentosi, SIMIFF), enrolling 23 hospitals from January 2009 through December 2011. All new FFI episodes, diagnosed by trained clinicians from mycology and radiology listings and/or histopatology reports, were prospectively collected by the national Coordinating Center (Laboratory of Mycology, Department of Biomedical Sciences and Human Oncology, University of Bari). The diagnostic and therapeutic management followed local practices. Dedicated medical personnel from each unit or hospital were trained in quality-controlled data collection. For each selected case, the hospitals had to complete a report form, including demographic characteristics, underlying disease, predisposing factors, prior antifungal treatment, microbiological, histological and imaging investigations, therapeutic approach and outcome at the 90th day after diagnosis. Every form was sent to the Coordinating Center, where an independent scientific advisory board met periodically to review the data collection procedures and review any diagnosis of infection.

The study was approved by the Ethics Committee of the Azienda Ospedaliero–Universitaria Policlinico of Bari, Italy, and by the institutional review board of each participating center, as appropriate. Registered data were managed in accordance with the Italian data protection laws (privacy law).

### Definitions

Only *proven*/*probable* cases were enrolled in this study. The classification of each case was defined by the European Organization for Research and Treatment of Cancer/Mycoses Study Group (EORTC/MSG) [19], with the following modifications:

1. patient enrollment regardless of underlying disease;
2. if diagnosis was made by histology with attainment of large and non-septate hyphae, the case was classified as *proven* mucormycosis, whereas observation of branched septate hyphae was considered generically diagnostic for hyalohyphomycosis.

In addition, pulmonary aspergilloma (PA) was classified as *proven* when specific antibodies were associated with a positive culture of *Aspergillus* species in patients with a solid rounded mass within a pulmonary cavity visualized on a computed tomography (CT) scan. In the absence of positive culture, the patients were classified as having *probable* PA [20].

The infection was defined as multiple when the patient had evidence of infection in >1 anatomic site and as disseminated when the infection involved >1 non-contiguous site reflecting hematogenous spread. Mixed infection was defined as the infection being caused by different mold genera.

Breakthrough infection was defined as the occurrence of *probable*/*proven* fungal infection while on prophylaxis with antifungals generally effective against mold for at least 7 days. Neutropenia was defined as an absolute neutrophil count of <1000 PMN/mm^3^ (moderate count, 500–1000 PMN/mm^3^; severe, 100–500 PMN/mm^3^; profound, ≤100 PMN/mm^3^).

Mortality was considered attributable (AM) to FFIs if the patients died with microbiological, histological, or clinical evidence of an active FFI and if other potential causes of death could be excluded by the responsible physician [21]. The crude mortality was defined as the ratio of deaths to the total number of enrolled patients.

### Statistical analysis

Univariate analysis was performed using the Chi square test (Fischer’s exact test) and the Mann–Whitney *U* (Student’s *t* test) when appropriate. Multivariate analysis, using logistic regression analysis, identified variables predicting mortality. The adjusted odds ratios (ORs) and 95 % confidence intervals (CIs) were calculated. Survival curves were prepared using the Kaplan–Meier method, and univariate survival distributions were compared using the Log rank test. In addition, Cox proportional hazard univariate models were performed to assess the survival difference between different FFIs.

Statistical analyses were performed with SPSS 10 for Mac OS X (SPSS Inc., Chicago, IL, USA). All of the tests were two-tailed, and statistical significance was defined as *p* < 0.05.

## Results

During a 3-year surveillance, 232 FFIs (83 *proven* and 149 *probable*) were documented: 113 HAEs and 119 non-HAEs (Table 1). The overall female/male ratio was 1.0:1.4, and the mean age was 51.14 ± 20.67 years (range 16–90 years).

**Table 1:** Clinical characteristics of 232 enrolled patients

Underlying disease/condition	No.	%
Hematological patients	113	48.7
Hematological malignancy^a^		
AML	63	55.7
ALL	19	16.8
NHL	12	10.6
CLL	8	7.1
MM	8	7.1
CML	2	1.8
HD	1	0.9
Non-hematological patients	119	51.3
Lung disease	28	23.5
COPD	13	76.4
Tuberculosis	9	32.1
Cystic fibrosis	2	7.1
Idiopathic fibrosis	2	7.1
Pulmonary emphysema	1	3.6
Pleuritis	1	3.6
Solid cancer	20	16.8
Lung	9	45.0
Intestine	5	25.0
Brain	3	15.0
Liver	1	5.0
Mouth	1	5.0
Heart	1	5.0
Organ transplantation	14	11.7
Lung	5	35.7
Kidney	5	35.7
Liver	4	28.6
Trauma	13	10.9
Surgery	9	7.6
Diabetes	8	6.7
HIV/AIDS	8	6.7
Autoimmune disorder	7	5.9
Cirrhosis	4	3.4
Burn	3	2.5
Renal disease	2	1.7
Other^b^	3	2.5

*AML* acute myeloid leukemia, *ALL* acute lymphoid leukemia, *CLL* chronic lymphocytic leukemia, *CML* chronic myeloid leukemia, *NHL* non-Hodgkin’s lymphoma, *HD* Hodgkin’s disease, *MM* multiple myeloma, *COPD* chronic obstructive pulmonary disease

^a^Thirty-one patients underwent transplantation are included in this group

^b^Aplastic anemia (*n* = 1); chronic granulomatous disease (*n* = 1); no risk factor (*n* = 1)

Aspergillosis was the most common fungal infection (71.1 %), followed by fusariosis (9.1 %), mucormycosis (6.9 %) and scedosporiosis (5.1 %). FFI localization was principally pulmonary (78.4 %), and central nervous system involvement (5.6 %) was always associated with other sites (lungs and orbito-sinus; Tables 2, 3).

**Table 2:** Filamentous fungal infections in 232 patients (166 with positive culture and/or PCR test)

Infection type and/or etiological agents	HAEs (*n* = 113)	Non-HAEs (*n* = 119)	All patients (*n* = 232)
Invasive aspergillosis, *n* (%)	86 (76.1)	67 (56.3)	153 (65.9)
*Aspergillus fumigatus*	18	28	46
*A*. *flavus*	14	12	26
*A*. *niger*	1	6	7
*A*. *terreus*	2	3	5
*A*. *flavipes*	1	–	1
*A*. *nidulans*	1	–	1
>2 species	1	3	4
Unspecified^a^	48	15	63
Aspergilloma, *n* (%)	–	12 (10.1)	12 (5.2)
*Aspergillus fumigatus*	–	6	6
*A*. *flavus*	–	2	2
*A*. *niger*	–	1	1
*A*. *terreus*	–	1	1
Unspecified^b^	–	2	2
Fusariosis, *n* (%)	11 (9.7)	10 (8.4)	21 (9.0)
*Fusarium solani*	3	6	9
*F*. *dimerum*	–	1	1
*F*. *proliferatum*	1	–	1
*F*. *verticilloides*	1	–	1
*Fusarium* spp	6	3	9
Mucormycosis^c^, *n* (%)	7 (6.2)	9 (7.6)	16 (6.9)
*Rhizopus oryzae*	3	3	6
*Mucor circinelloides*	0	2	2
*Absidia corymbifera*	1	–	1
*Rhizomucor pusillus*	1	–	1
*Mucor* spp	1	2	3
*Rhizopus* spp	1	1	2
*Scedosporium apiospermum*, *n* (%)	–	6 (5.0)	6 (2.6)
*Paecilomyces lilacinus*, *n* (%)	–	3 (2.5)	3 (1.3)
*Alternaria alternata*, *n* (%)	1 (0.9)	2 (1.6)	3 (1.3)
*Trichoderma viride*, *n* (%)	2 (1.8)	–	2 (0.9)
*Acremonium* spp, *n* (%)	–	1 (0.8)	1 (0.4)
*Exophiala dermatitidis*, *n* (%)	–	1 (0.8)	1 (0.4)
Hyalohyphomycosis, *n* (%)	6 (5.3)	4 (3.4)	10 (4.3)
Mixed infection^d^, *n* (%)	–	4 (3.4)	4 (1.7)

*HAEs* hematological patients, *non-HAE*: non-hematological patients

^a^Includes patients with invasive aspergillosis diagnosed by galactomannan assay

^b^Includes patients with aspergilloma diagnosed by serological test

^c^In one patient the diagnosis was made by observation at histology

^d^ *M*. *circinelloides* + *Fusarium* spp; *M*. *circinelloides* + *S*. *apiospermum*; *Mucor* spp + *A*. *flavus*; *R*. *oryzae* + *A*. *nidulans*

**Table 3:** Clinical signs and symptoms according to the main sites of fungal infection

Sign/symptom	HAEs (*n* = 113)	Non-HAEs (*n* = 119)	All patients (*n* = 232)
Pulmonary localization, *n* (%)	94	89	183
Fever	73 (77.6)	54 (60.7)	127 (69.4)
Dyspnea	28 (29.8)	43 (48.3)	71 (38.8)
Cough	16 (17.0)	30 (33.7)	46 (25.1)
Chest Pain	11 (11.7)	10 (11.2)	21 (11.5)
Hemoptysis	7 (7.4)	6 (6.7)	13 (7.1)
Blood, *n* (%)	10	6	16
Fever	9 (90.0)	4 (66.7)	13 (81.2)
Orbito-sinus localization, *n* (%)	8	8	16
Fever	7 (87.5)	4 (50.0)	11 (68.7)
Facial pain/edema/palsy	5 (62.5)	1 (12.5)	6 (37.5)
Headache	–	3 (37.5)	3 (18.7)
Orbital cellulitis	1 (12.5)	2 (25.0)	3 (18.7)
Necrotic lesions	2 (25.0)	1 (12.5)	3 (18.7)
Ophthalmoplegia/Rhinorrhea	2 (25.0)	1 (12.5)	3 (18.7)
Central nervous system involvement, *n* (%)	8	5	13
Fever	8 (100.0)	2 (40.0)	10 (76.9)
Headache	2 (25.0)	2 (40.0)	4 (30.7)
Blurred/double vision	2 (25.0)	2 (40.0)	4 (30.7)
Apathy/confusion	–	2 (40.0)	2 (15.3)
Facial palsy/ophthalmoplegia	2 (25.0)	1 (20.0)	3 (23.1)
Skin and soft tissue involvement, *n* (%)	7	5	12
Lesion (papule, vesicle, ulcer, necrosis)	3 (42.8)	5 (100.0)	8 (66.7)
Fever	3 (42.8)	2 (40.0)	5 (41.7)
Pain	1 (14.3)	2 (40.0)	3 (25.0)
Hypothermia	–	1 (20.0)	1 (8.3)
Eye involvement, *n* (%)	0	10	10
Pain	–	7 (70.0)	7 (70.0)
Hypopyon	–	5 (50.0)	5 (50.0)
Photophobia	–	3 (30.0)	3 (30.0)
Corneal ulcer	–	2 (20.0)	2 (20.0)
Eyelid swelling	–	2 (20.0)	2 (20.0)
Fever	–	1 (10.0)	1 (10.0)
Abdominal involvement, *n* (%)	4	3	7
Pain/diarrhea	4 (100.0)	3 (100.0)	7 (71.4)
Fever	3 (75.0)	2 (66.7)	5 (71.4)
Asymptomatic patients, *n* (%)	–	3	3

*HAEs* hematological patients, *non-HAE* non-hematological patients

**Fig. 1:**
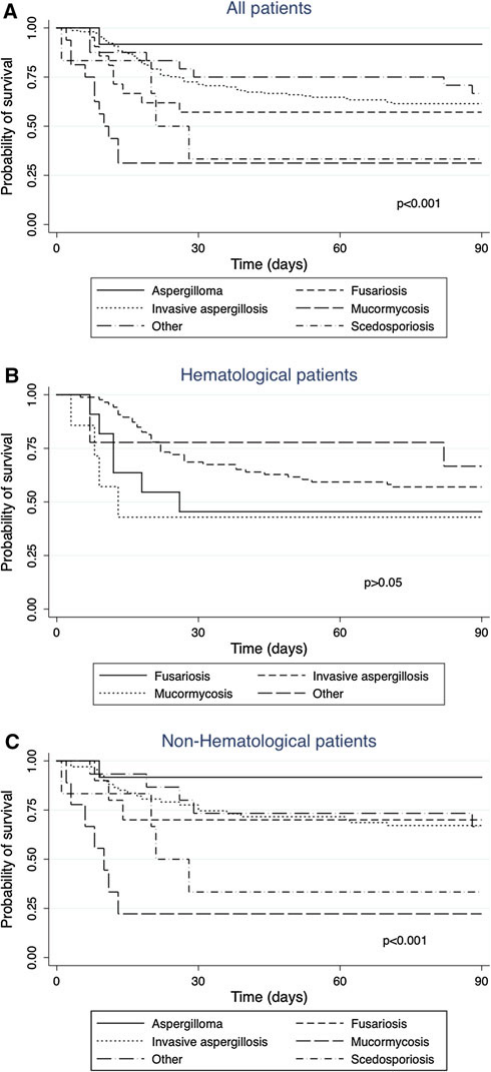
Ninety-day survival according to filamentous fungal infections type in all enrolled patients (**a**), in hematological patients (**b**) and in non-hematological patients (**c**)

The crude mortality rate was 39.6 % (AM = 38 %), and it varied significantly as a function of the type of infection (*p* < 0.001; Fig. 1a). The patients with mucormycosis exhibited the worst survival (median failure time for death, 8 days; mortality rate, 75 %). Mortality was higher for mucormycosis compared with aspergilloma (hazard ratio [HR] 12.2; 95 % CI 1.6–95.2; *p* = 0.017), and invasive aspergillosis (HR 3.5; 95 % CI 1.8–6.7; *p* < 0.001). Patients with scedosporiosis appeared to have stronger mortality (HR 10.3; 95 % CI 1.1–93; *p* = 0.038) compared to patients with aspergilloma.

Moreover, the patients with central nervous system involvement appeared to have a higher mortality compared with other sites (median failure time for death, 13 days; mortality rate, 91.7 %; *p* < 0.001).

### Hematological patients

Among the 113 HAEs, acute leukemia was the leading malignancy (*n* = 82, 63 acute myeloid leukemia and 19 acute lymphoid leukemia patients), followed by lymphoproliferative disorders (*n* = 28; Table 1). Thirty-one patients (27.4 %) received an HSCT: 30 allogeneic and one autologous HSCT. One hundred and one patients were neutropenic (89.3 %), and of these, 64 (63.4 %) had an absolute neutrophil count <100 PMN/mm^3^. The mean duration of neutropenia was 25.1 ± 17 days (range 6–75 days). Seventy-eight patients (69 %) received cytotoxic chemotherapy. Corticosteroids were previously administered to 42 patients (37.1 %), and 24 patients (21.2 %) received immunosuppressive therapies (19 HSCT and 5 no-HSCT). Graft versus host disease (GVHD) was documented in 14 patients (12.4 %), and 65 % of these patients had acute GHVD. Eight patients (7.1 %) had diabetes, and 7 HSCT patients (6.2 %) had a co-infection with cytomegalovirus.

Invasive aspergillosis (IA; *n* = 86; 76.1 %) was the most common disease (Table 2) with *Aspergillus* spp isolated in 38 patients (44.1 %). The ratio of aspergillosis/no-*Aspergillus* infection was 3:1. Fusariosis emerged as the second FFI (*n* = 11; 9.7 %), followed by mucormycosis (*n* = 7; 6.2 %).

Pulmonary involvement was evident in 94 patients (83.2 %; Table 3), and it tended to be more common among the IA patients than among those with other mycoses (84/86 cases vs. 10/27 cases; *p* < 0.001). Disseminated disease was observed in 10.6 % of cases (*n* = 12; seven aspergillosis and five fusariosis).

Regarding the diagnostic methods (Table 4), the diagnosis was made by microbiological culture in 49 patients (43.4 %) and by histology in six patients (5.3 %). Both methods returned positive results in nine other patients (7.9 %). The galactomannan (GM) assay was positive in 82 IA patients (95.3 %), and this assay produced the only positive microbiological data for 48 patients (58.5 %).

**Table 4:** Diagnostic methods used for diagnosis of different types of filamentous fungal infections (FFIs)

Diagnostic method^a^	Aspergillosis		Fusariosis		Mucormycosis		Scedosporiosis	Mixed Infection	Other FFIs		All FFIs	
	HAEs *n* = 86	Non-HAEs *n* = 79	HAEs *n* = 11	Non-HAEs *n* = 10	HAEs *n* = 7	Non-HAEs *n* = 9	Non-HAEs *n* = 6	Non-HAEs *n* = 4	HAEs *n* = 9	Non-HAEs *n* = 11	HAEs *n* = 113	Non-HAEs *n* = 119
Culture	38 (44.2)^b^	62 (78.5)	10 (90.9)	10 (100)	7 (100)	8 (88.9)	6 (100)	4 (100)	3 (33.3)	7 (63.6)	58 (51.3)	97 (81.5)
Direct examination	21 (24.4)	24 (30.4)	6 (54.5)	5 (50)	4 (57.1)	7 (77.8)	3 (50)	4 (100)	1 (11.1)	3 (27.3)	32 (28.3)	46 (38.6)
Galactomannan test	82 (95.3)	38 (48.1)	–	–	–	–	–	–	1 (11.1)	2 (18.2)	83 (73.4)	40 (33.6)
β-D-Glucan test	1 (1.2)	–	3 (27.3)	1 (10)	–	–	2 (33.3)	–	2 (22.2)	2 (18.2)	6 (5.3)	5 (4.2)
Polymerase chain reaction	5 (5.8)	1 (1.3)	1 (9.1)	–	–	–	–	–	–	–	6 (5.3)	1 (0.8)
Serological test	–	10 (12.6)	–	–	–	–	–	–	–	–	–	10 (8.4)
Histology	4 (4.6)	5 (6.3)	2 (18.2)	–	3 (42.8)	6 (66.7)	2 (33.3)	2 (50)	6 (66.7)	4 (36.4)	15 (13.3)	19 (16)
Radiology												
Chest radiograph	44 (51.2)	64 (81.0)	1 (9.1)	2 (20)	2 (28.6)	3 (33.3)	2 (33.3)	1 (25)	4 (44.4)	4 (36.4)	51 (45.1)	76 (63.9)
Computed tomography scan	65 (75.6)	34 (43.0)	4 (36.4)	1 (10)	5 (71.4)	6 (66.7)	3 (50)	2 (25)	4 (44.4)	3 (27.3)	78 (69.0)	49 (41.2)
Magnetic resonance imaging	3 (3.5)	–	1 (9.1)	–	2 (28.6)	3 (33.3)	1 (16.7)	–	1 (11.1)	–	7 (6.2)	4 (3.4)
Slit-lamp examination	–	–	–	3 (30)	–	–	2 (33.3)	–	–	2 (18.2)	–	7 (5.9)
Fundus examination	–	–	–	2 (20)	–	–	2 (33.3)	–	–	2 (18.2)	–	6 (5.0)

*HAEs* hematological patients, *non-HAE* non-haematological patients

^a^Diagnostic methods were not mutually exclusive: >1 tests might have been used for the FFI diagnosis

^b^Number in parentheses, percent

Overall, 101 patients were subjected to different radiographic procedures, alone or in combination. Regarding pulmonary involvement, CT scans revealed several patterns such as halo signs (33/72; 45.8 %), areas of consolidation (20/72; 27.8 %), and nodules (14/72; 19.4 %). Cavitated nodules were less common (3/72; 4.2 %). Regarding sinus involvement, the radiographic findings indicated mucosal thickening and opacification, whereas the head imaging revealed cerebral intraparenchymal lesions.

Seventy-five patients (66.4 %) received antifungal prophylaxis (mean treatment 34 days, range 2–165). Fluconazole (*n* = 56; 74.6 %) was the most used drug, followed by posaconazole (*n* = 12; 16 %) and itraconazole (*n* = 7; 9.3 %). FFIs presented as breakthrough infections in 18 patients (15.9 %). Antifungal treatment was empirically started in 44 patients (38.9 %).

After the FFI diagnosis, antifungal treatment was employed in 109 (96.4 %) episodes, and four patients (three aspergillosis and one hyalohyphomycosis) were not treated because of pre-diagnosis death. Eighty-six patients (78.9 %) received antifungal monotherapy, most commonly with voriconazole (*n* = 60), liposomal amphotericin B (L-AmB) (*n* = 16) and caspofungin (*n* = 7). Combination therapy was administered in 23 cases (21.1 %), generally voriconazole plus caspofungin (*n* = 16). In addition to antifungal therapy, curative surgical interventions (lobectomies, excisional biopsies and debridement) were conducted in nine patients. The crude mortality rate was 44.2 %.

Univariate analysis revealed that the following variables had a significant influence on death (Table 5): profound neutropenia, corticosteroid therapy, and cytomegalovirus infection. In the multivariate analysis, the parameters that were independently associated with an increased risk of death were profound neutropenia (OR 4.6, 95 % CI 1.3–16.6) and corticosteroid therapy (OR 13.6, 95 % CI 3.9–47.5). The 90-day survival rate varied considerably based on the type of infection, and mucormycoses were associated with a worse survival (median failure time for death 8.5 days; mortality rate 57.1 %; Fig. 1b). The HR for mucormycosis compared with invasive aspergillosis was 3.2 (95 % CI 1.6–6.3; *p* = 0.001).

**Table 5:** Variables associated with deaths within 90 days after filamentous fungal infections diagnosis for hematological patients (HAEs) and non-hematological patients (HAEs)

Variable^a^	HAEs (*n* = 113)				
	Univariate analysis		Multivariate analysis		
	Alive *n* = 63 (%)	Died *n* = 50 (%)	*p* value	OR (95 % CI)	*p* value
Profound neutropenia	28 (44.4)	36 (72.0)	0.011	4.6 (1.3–16.6)	0.018
Corticosteroids therapy	10 (15.9)	32 (64.0)	<0.001	13.6 (3.9–47.5)	<0.001
Cytomegalovirus infection	1 (1.6)	6 (12.0)	0.022	–	–

Variable^b^	Non-HAEs (*n* = 119)				
	Alive *n* = 77 (%)	Died *n* = 42 (%)	*p* value	OR (95 % CI)	*p* value
Lung diseases	23 (29.9)	5 (11.9)	0.027	–	–
Mechanical ventilation	7 (9.1)	17 (40.5)	<0.001	6.7 (2.3–19.2)	<0.001
Mucormycosis	2 (2.6)	7 (16.7)	0.005	–	–
Multiple infection	4 (5.2)	10 (23.8)	0.002	4.7 (1.1–18.7)	0.03

Only the statistically significant are shown (*p* < 0.05)

^a^Variables tested in the univariate analysis included age, gender, underlying diseases (hematopoietic stem cell transplantation, acute mieloid leukemia), neutropenia (moderate, severe, profound), corticosteroids therapy, cytomegalovirus infection, presence or absence of graft-versus-host disease, invasive aspergillosis, sites of infection (lung only and multiple infection), surgical treatment and certainty of diagnosis

^b^Variables tested in the univariate analysis included age, gender, underlying diseases (lung disease, solid cancer, solid organ transplantiation, trauma, diabetes), corticosteroids therapy, mechanical ventilation, antifungal prophylaxis, invasive aspergillosis, fusariosis, mucormycosis, sites of infection (lung only and multiple infection), surgical treatment and certainty of diagnosis

### Non-hematological patients

Among the 119 non-HAEs, lung disease, solid cancer, organ transplantation and trauma were the most common underlying disease/risk factors associated with FFI (Table 1). Other predisposing conditions were prolonged corticosteroid treatment (52.9 %) and prolonged ICU stay associated with ventilation and/or parental nutrition (20.1 %). Diabetes was the sole predisposing factor in eight patients (6.7 %), whereas in another 19 cases (15.9 %), diabetes was combined with other underlying conditions, such as COPD and cancer.

Aspergillosis was the predominant clinical entity (Table 2): IA in 67 patients (56.3 %) and PA in 12 (10.1 %) patients. Positive cultures of *Aspergillus* species were obtained in 62 cases (78.4 %), and *A*. *fumigatus* and *A*. *flavus* were the main species identified. Among the other infections, fusariosis occurred in 10 patients (8.4 %), followed by mucormycosis (*n* = 9; 7.6 %) and scedosporiosis (*n* = 6; 5 %).

The lower respiratory tract was the most commonly involved site (Table 3), with 83 patients (69.7 %) having only lung infection. Extrapulmonary involvement occurred in 31 patients (26.1 %, mostly eye and paranasal sinuses), and disseminated disease occurred in nine cases (7.5 %).

The diagnosis (Table 4) was mostly performed by positive microbiological culture (*n* = 97; 81.5 %), of which 36 (37. %) were biopsy samples. In terms of imaging, in patients with IA, pulmonary segmental areas of consolidation were the most frequent finding in CT scans and on plain radiographs. Evidence of pulmonary intra-cavitary mass suggestive of a fungal ball was observed in patients with aspergilloma and *Scedosporium* infection. Regarding the ocular mycoses (four fusariosis, two paecilomycosis, two scedosporiosis, one alternariosis and one mucormycosis), slit-lamp examination led to infection suspicion each time that it was performed, revealing chemosis, corneal edema and anterior chamber inflammation. The fundus examination indicated vitreous opacity and white punctate infiltrates. For the patients with cerebral infection, the imaging patterns included hypodense foci and scattered lesions of various sizes, whereas signs of diffuse sinusitis were detected in sinusal disease.

Before the diagnosis, fluconazole prophylaxis (mean treatment 11.6 days, range 1–40) was employed only in 10 (8.4 %) patients. An empirical therapy was given in 26 patients (21.8 %; azoles in 15, L-AmB in 9 and caspofungin in two patients).

After the diagnosis, antifungal therapy was administered to 114 patients (95.8 %), combined with curative surgical intervention in 20 patients. Five patients were not treated because of early death. Monotherapy was prescribed for 100 patients (87.7 %), and voriconazole was administered for the majority of patients with aspergillosis (*n* = 50/64; 78.1 %), fusariosis (6/10; 60 %) and scedosporiosis (3/5; 60 %). L-AmB was used in seven cases of mucormycosis (77.8 %).

Combination treatment was used in 14 patients (12.3 %), and L-AmB with an azole was the most frequently administered combination.

The crude mortality rate was 35.3 %. Table 5 shows the variables that significantly affected mortality according to univariate and multivariate analyses. Multiple logistic regression revealed that undergoing mechanical ventilation (OR 6.7, 95 % CI 2.3–19.2) and the presence of multiple infection (OR 4.7, 95 % CI 1.1–18.7) were factors that were independently associated with poor outcome.

The 90-day survival rate varied significantly according to the type of infection (*p* < 0.001), ranging from 22.2 % for patients with mucormycosis to 91.7 % for those with aspergilloma (Fig. 1c). Mortality was higher for mucormycosis compared with aspergilloma (HR 12.2; 95 % CI 1.6–95.2; *p* = 0.017), and invasive aspergillosis (HR 3.6; 95 % CI 1.7–7.5; *p* = 0.001).

### Comparison between hematological and non-hematological patients

The results of comparative analyses of clinical and biological findings are reported in Table 6. The distribution of *probable* and *proven* FFIs was not similar between the two groups: *proven* infections occurred more frequently in non-HAEs, whereas *probable* FFIs were correlated with HAEs. The rate of positive GM antigenemia was higher for HAEs. The only significant difference in the thoracic CT findings was the halo sign, which was more common in HAEs.

**Table 6:** Comparison of clinical and biological findings between hematological patients (HAEs) and non-hematological patients (HAEs)

Variable	HAE *n* = 113	Non-HAE *n* = 119	*p* value
Certainty of diagnosis, *n* (%)			0.010
Proven	31 (27.4)	52 (43.7)	
Probable	82 (72.6)	67 (56.3)	
Underlying conditions, *n* (%)			
Neutropenia	101 (89.4)	9 (7.6)	<0.001
Corticosteroids therapy	42 (37.2)	63 (52.9)	0.016
Prolonged antibiotic therapy	51 (45.1)	47 (39.5)	0.385
Immunosuppressive therapy	24 (21.2)	15 (12.6)	0.079
Cytomegalovirus infection	7 (6.2)	1 (0.8)	0.025
Diabetes	8 (7.1)	27 (22.7)	0.001
Mechanical ventilation	2 (1.8)	24 (20.2)	<0.001
Surgery	2 (1.8)	21 (17.6)	<0.001
Site of localization, *n* (%)			
Lung	94 (83.2)	89 (74.8)	0.117
Eye	0	10 (8.4)	0.002
Blood	8 (7.1)	5 (4.2)	0.341
Multiple	16 (14.1)	14 (11.8)	0.587
Mean time between symptoms and diagnosis, no. days	11.06 (1–57)	13.65 (0–65)	0.900
Fever, *n* (%)	103 (91.1)	69 (58.0)	<0.001
GM antigenemia positive for invasive aspergillosis, *n* (%)	82/86 (95.3)	38/79 (48.1)	<0.001
Mean time between symptoms and therapy, no. days	12 (0–57)	14.3 (0–65)	0.147
Antifungal prophylaxis, *n* (%)	75 (66.4)	10 (8.4)	<0.001
Empirical therapy, *n* (%)	44 (38.9)	26 (21.8)	0.005
Combination therapy, *n* (%)	23/109 (21.1)	14/114 (12.3)	0.076
Computed tomography signs recorded in patients with pulmonary involvement, *n* (%)			
Halo sign	33/94 (35.1)	10/89 (11.2)	<0.001
Areas of consolidation	21/94 (22.3)	14/89 (15.7)	0.256
Nodules	14/94 (14.9)	10/89 (11.2)	0.464
Crude mortality, *n* (%)	50 (44.2)	42 (35.3)	0.163

The rate of underlying conditions in HAEs differed from that in non-HAEs: neutropenia and cytomegalovirus infection were more common in HAEs, whereas corticosteroid therapy, surgery, diabetes mellitus and mechanical ventilation occurred more frequently in non-HAEs. Fever was a prominent feature among the HAEs.

The proportions of lung diseases, fungemia and infection in multiple sites were similar in both groups, but ocular mycoses were exclusively found among the non-HAEs. No significant difference was detected in mortality between the two groups. HAEs had a shorter median survival time (13.0 vs. 18.5 days), but this difference was not significant. Antifungal prophylaxis and empirical therapy were mainly employed in HAEs, and no significant difference in combination therapy was observed.

## Discussion

This study reports the first dataset in Italy that captures and displays information on FFIs in HAEs and non-HAEs. Although this registry cannot yield any information on incidence rate, given the lack of an FFI risk-population denominator, it can be considered representative of all FFI cases in Italy.

The first finding emerging between HAEs and non-HAEs is the different distribution of *proven* and *probable* FFIs (*p* = 0.010). HAEs are correlated with *probable* cases (72.6 vs. 27.4 %), which suggests a different diagnostic work-up. FFI diagnosis was mainly based on clinical and antigen investigations. Notably, the majority of IAs were due to unknown *Aspergillus* spp (55.8 %) because GM results were promptly obtained in these patients. Therefore, the possibility of obtaining an early and non-invasive diagnosis led the hematologists to neglect pursuing a diagnosis of *proven* FFI based on invasive diagnostic tools, which are difficult to use in these patients. [24]. In contrast, we observed a high proportion of *proven* diseases (43.7 %) in the non-HAE patient setting, where the clinicians often do not apply the criteria for *probable* cases [19] and are encouraged to use a more intensive diagnostic approach to overcome the degree of probability because of the well-known poor performance of the GM test [25, 26]. In fact, the GM levels are significantly more reliable in patients with neutropenia [26], and the low rate of neutropenic patients among the non-HAE group could justify the modest percentage of GM positivity in these patients compared with the HAE patients (48.1 vs. 95.3 %, *p* < 0.001). Moreover, prophylaxis with drugs effective against mold can interfere with GM performance [26]. In HAEs, the high yield of GM is most likely because at the time of our survey, posaconazole prophylaxis was rarely performed [27], although recent evidence appears to confirm the importance of the GM assay in the work-up of patients on posaconazole prophylaxis [28]. Currently, the risk of poor GM assay performance in patients under posaconazole prophylaxis [29] should be kept in mind.

Prophylaxis was performed more frequently in HAEs, with a preference regarding the type of azole favoring fluconazole, which may explain the prevalence of empirical therapy in the same patient setting, given the known lack of effectiveness of fluconazole against many *Candida* spp and molds.

An analysis of the type of infection reveals a clear prevalence of aspergillosis in both types of patients but particularly in HAEs (76.1 vs. 56.3 %, *p* = 0.102). These results are in agreement with data reported in the literature [12, 30] indicating aspergillosis primacy, regardless of the patient type. Although lung diseases (i.e., COPD and cancer) constituted the prevalent baseline condition of the non-HAEs, pulmonary involvement did not differ between the HAEs and non-HAEs (83.2 vs. 74.8 %, *p* = 0.117), confirming the crucial role of lungs in FFI-risk patients [31].

Overall, the mortality rate was 39.6 %, and no distinct difference between HAEs and no-HAEs (42.2 vs. 35.3 %, *p* = 0.163) was observed, indicating that the FFIs are severe diseases, most likely because the causative agents have developed strategies for the recognition and/or eradication of the immune defenses [32, 33]. For non-HAEs, the present mortality rate was lower than those reported in previous studies [8, 10, 12], suggesting an increase in clinician awareness of the risk of FFI in these patients.

Regarding non-*Aspergillus* infections, the data reported in the literature reveal a high mortality rate, most likely because these infections are difficult to diagnose and these fungi have a tendency to be disseminated and resistant to most of the available antifungal agents [34]. In our study, the survival rate varied according to the FFI. A trend toward worse survival was observed among patients with mucormycosis in HAEs and in non-HAEs, although survival in HAEs did not significantly differ according to the type of infection. This tendency might be dependent on the different types of underling clinical conditions of the HAEs, irrespective of the subsequent FFI diagnoses.

Finally, some differences concerning host variables and comorbidities were observed in the two groups. Our findings confirm the importance of the depth and length of neutropenia (particularly correlated with conventional chemotherapy) and of cytomegalovirus infection as a factor contributing to the development of FFIs in HAEs [9, 35]. In contrast, corticosteroid treatment was the main underlying condition in the non-HAE cohort. It is well recognized that corticosteroids induce complex immune dysregulation that impairs neutrophil and macrophage function. In addition, the anti-inflammatory properties of glucocorticoids usually blunt the signs and symptoms of FFI, and the patients may not present raised temperatures [36]. These factors may explain the absence of fever in almost half of the non-HAEs.

In conclusion, the FFI epidemiological and clinical data were not identical in the two groups of patients. On the other hand, having diagnosed the FFI cases in the non-HAE patients predominantly on the basis of culture, and in the HAE patients on the basis of GM (as microbiological criteria), it may appear obvious that this different diagnostic management may lead to a presumptive diagnostic delay in non-HAE patients. Nevertheless, any effort should be made to overcome the risk of delayed antifungal treatments avoiding a fever-driven diagnostic approach (enforcing CT scan examination and non-culture antigen-based diagnostics) and using an empirical strategy in case of fever.
